# Calcitonin Gene-Related Peptide (CGRP) and Cluster Headache

**DOI:** 10.3390/brainsci10010030

**Published:** 2020-01-06

**Authors:** Andrea Carmine Belin, Caroline Ran, Lars Edvinsson

**Affiliations:** 1 Department of Neuroscience, Karolinska Institutet, 171 77 Stockholm, Sweden; andrea.carmine.belin@ki.se; 2 Department of Clinical Sciences, Division of Experimental Vascular Research, Lund University, 221 84 Lund, Sweden; 3 Department of Clinical Experimental Research, Glostrup Research Institute, Rigshospitalet Glostrup, 2600 Glostrup, Denmark

**Keywords:** neurovascular, receptor, vasodilator, antibodies, genetics

## Abstract

Cluster headache (CH) is a severe primary headache with a prevalence of 1/1000 individuals, and a predominance in men. Calcitonin gene-related peptide (CGRP) is a potent vasodilator, originating in trigeminal neurons and has a central role in CH pathophysiology. CGRP and the CGRP receptor complex have recently taken center stage as therapeutic targets for primary headaches, such as migraine. Multiple CGRP and CGRP receptor monoclonal antibodies, as well as small molecule antagonists (gepants) are on their way constituting a new frontier of migraine and possibly CH medication. During a CH attack, there is an activation of the trigeminal-autonomic reflex with the release of CGRP, and inversely if CGRP is administered to a CH patient in an active disease phase, it triggers an attack. Increased levels of CGRP have been found in ipsilateral jugular vein blood during the active phase of CH. This process is hypothesized to have a key role in the intense pain perception and in the associated distinctive vasodilation. So far, clinical tests of CGRP antibodies have been inconclusive in CH patients. This review summarizes the current state of knowledge on the role of CGRP in CH pathology, and as a target for future treatments.

## 1. Introduction

Cluster headache (CH) is a severe trigeminal autonomic cephalalgia (TAC), where symptoms include recurrent headaches of extreme intensity, accompanied by autonomic symptoms and restlessness. TACs are a group of primary headaches that occur with pain in the trigeminal nerve area on one side of the head and ipsilateral symptoms in autonomic systems, such as drooping eyelids and eye redness [1]. In addition to CH, TACs include paroxysmal hemicrania, short-lasting unilateral neuralgiform headache attacks with conjunctival injection and tearing (SUNCT), short-lasting unilateral neuralgiform headache attacks with cranial autonomic symptoms (SUNA), and hemicrania continua [1]. TACs are differentiated by the duration of the headaches and the frequency of recurrence. The prevalence of CH has been estimated to around 0.1–0.05%, consistently higher in males than females [2,3]. 85% of the CH patients have an episodic form, experiencing attack (active) periods and attack free (remission) periods of more than three months per year [1]. The remaining 15% suffer from a chronic form with short and few remission periods if any. CH is often described as the most painful disorder known to affect humans, and is sometimes referred to as “suicide headache” [4,5]. Although the first known description of CH was made by Dr. Nicolaas Tulp in the 17th century, no pharmacological treatment has yet been specifically developed for CH only [6]. Existing treatments for CH remain insufficient with varying degrees of pain relief and many side effects. CH and migraine share certain phenotype properties and are, to some extent, treated with the same drugs. Substances with vasoconstrictive properties like sumatriptan, a serotonin receptor agonist for 5-HT1B and 5-HT1D and oxygen are mainly used for abortion of an acute CH attack. Devices, such as the noninvasive vagus nerve stimulation (nVNS) are approved by the US Food and Drug Administration (FDA) for the acute treatment of attacks in episodic CH. nVNS is additionally used as a prophylactic treatment for both episodic and chronic CH. Verapamil, a calcium channel blocker, is the most common reported used preventive treatment for CH today, but lithium, prednisolone and topiramate are used as well.

The neuropeptide calcitonin gene-related peptide (CGRP) is a potent vasodilator produced in both central and peripheral neurons [7,8,9]. CGRP was discovered in 1982 and is a member of the calcitonin family of peptides, also comprising calcitonin, amylin (AMY), and adrenomedullin (AM) [10,11,12]. CGRP exists in two forms in humans, α-CGRP and β-CGRP [13]. α-CGRP is a 37-amino acid long peptide, formed from the alternative splicing of the calcitonin related polypeptide alpha gene (CALCA) located on chromosome 11p15.2 [10]. The gene spans a region of about 6.3 kilo bases (kb) and comprises a total of 6 exons. The β-CGRP peptide is less studied. It differs from α-CGRP only in three amino acids, and is encoded by a separate gene in close proximity; calcitonin related polypeptide beta (CALCB) [13]. In this review, we will only refer to α-CGRP when describing CGRP.

Evidence has accumulated rapidly on the implication of CGRP in primary headache during the last 30 years, and resulted in the approval of a CGRP receptor antibody as a new preventive treatment for migraine in 2018 [14]. Monoclonal antibodies targeted against CGRP or its receptors are the first treatment developed specifically for headache since the release of triptans in 1991 [15]. These antibodies are also being investigated as a possible treatment for CH. Professor Lars Edvinsson was the first to describe CGRP in the trigeminal system and early pointed out that it was a breakthrough in understanding neural regulation of the cerebral circulation and associated with migraine pathophysiology [16]. CGRP was first suspected to be involved in CH pathophysiology in the late 1980s [17]. In relation to CH, CGRP originates in the trigeminal ganglion with sensory C- and Aδ-fibers that project to various parts of the face and head, as well as to intracranial structures [7,18,19]. It is now established that there is activation of the trigeminal-autonomic reflex with the release of CGRP during a CH attack, and that CGRP administered to a CH patient in an active disease phase triggers an attack [20,21]. In addition, it was early observed that CGRP levels were increased following induction of a CH attack by systemic administration of nitroglycerin [22].

Most studies concerning CGRP and headache has been focused on migraine [23]. In this review, we will summarize and highlight the involvement of CGRP in CH with regard to pathophysiology, genetics and future treatment.

## 2. CGRP Localization

**Figure 1:**
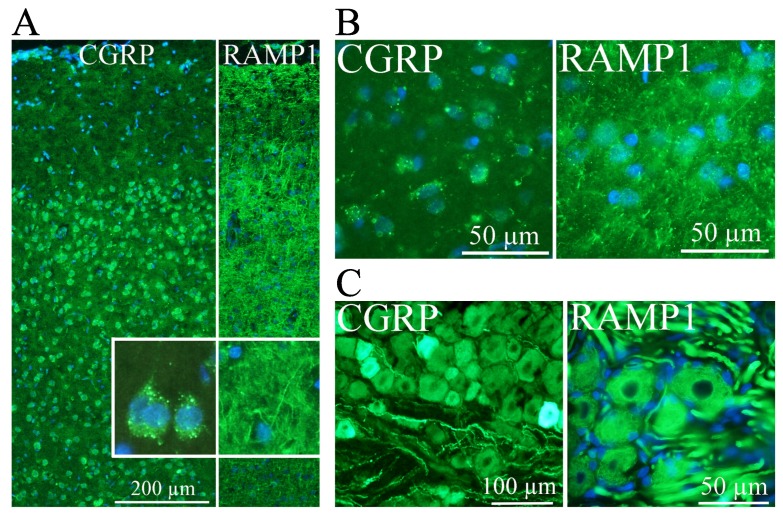
Calcitonin gene-related peptide (CGRP) and the CGRP receptor component receptor activity-modifying protein 1 (RAMP1) distribution using immunohistochemistry in rat brain, (modified from Warfvinge and Edvinsson, Cephalalgia 2019 [24]). Nuclei are stained blue with DAPI. In cortex (**A**), CGRP (left panel) is localized to neuronal cell bodies in cortical layers II-VI. Insert confirms the cytoplasmic location, and granular pattern of CGRP staining, as well as the lack of staining in neuronal processes. RAMP1 (right panel) was localized in processes and found to travel in all directions, horizontal, between layers or spanning through the entire gray matter. In the paraventricular hypothalamic nucleus (**B**), the distribution of CGRP and RAMP1 was similar; CGRP being localized to the cytoplasm of neurons, while RAMP1 was present in cellular processes. In the trigeminal ganglion (**C**), both CGRP and RAMP1 are observed in cell bodies. CGRP was localized to small and medium-sized neurons, and to thin neuronal processes. RAMP1, in contrast, was observed in larger neurons and thicker processes.

The first studies on CGRP localization were presented by means of a northern blot on RNA extracted from different cell lines and tissues, and determined that CGRP was predominantly expressed in the hypothalamus, while calcitonin was preferably expressed in the thyroid [10]. The localization of the CGRP peptide in neurons is a consequence of neuron specific expression of the proteins required for splicing exon 4 in CALCA mRNA [24]. Recent mapping of CGRP and CGRP receptors revealed an overall widespread expression in the rat brain (Figure 1), including migraine-related sites [25]. CGRP is actually one of the most abundant peptides in the nervous system, for review, see Van Rossum et al. [26]. However, using more precise methodology, it is now clear that CGRP and its receptor, composed of calcitonin receptor-like receptor (CALCRL) and receptor activity-modifying protein 1 (RAMP1), have a broad function across several tissues. CGRP positive neurons are distributed throughout both the central and peripheral nervous systems, and can, therefore, exhibit a range of biological effects on tissues, including gastrointestinal, respiratory, cardiovascular, urogenital, endocrine, sensory and central nervous systems [26,27,28,29,30,31,32,33,34,35].

CGRP is localized to regions of the nervous system that are key players in CH pathophysiology, like the hypothalamus (Figure 1B). Importantly, the posterior hypothalamus is activated during CH attacks [36,37]. In addition, many CH patients experience that their attacks occur at certain time points during the day, with a peak in frequency between 02:00 and 04:00 in the morning [38,39]. Last, the secretion of several hormones that are altered in CH, e.g., melatonin and cortisol, is under hypothalamic control [40]. In the hypothalamus, CGRP-positive neurons have been identified in the supraoptic nucleus, paraventricular nuclei and infundibular nuclei, and it has, therefore, been suggested that CGRP might influence the hypothalamo-pituitary axis [41]. In the trigeminal ganglion, a structure highly relevant in CH pathophysiology, both cell bodies and nerve fibers contain a number of messenger molecules, and CGRP is the one most frequently expressed in humans (Figure 1C) [42,43]. Immunohistochemical studies of rat and rhesus monkey have confirmed the expression of the CGRP receptor in neurons and glial cells in the trigeminal ganglion, and further showed that CGRP and its receptor were mainly expressed in distinct cells [43,44]. CGRP has been reported to modulate cytokine release from satellite glial cells, which surround the trigeminal ganglia neurons [45]. CGRP is expressed in C-fibers, while its receptor is expressed in Aδ-fibers, two types of fibers involved in different aspects of pain perception [19,46]. To transmit pain signals these fibers project both centrally via the brainstem trigeminal nucleus caudalis to the thalamus and higher cortical pain regions and to various cranial sites in the peripheral direction, such as blood vessels or dura mater [19,47,48,49].

Overall there is a rich CGRP innervation of the vascular system, which is the structural basis of the vasodilatory effect of CGRP. CGRP receptor immunoreactivity has been observed in the vessel wall of veins and arteries throughout the human body [50]. While the CGRP receptor is predominantly localized to the endothelial layer and the smooth muscle of the tunica media, CGRP vasodilation was found to be independent of the endothelium [51]. Of particular interest in headache, pathophysiology is the innervation of vasculature in the head, face and meninges. The carotid artery and its branches are receiving synaptic input from neurons in the trigeminal ganglion [35]. Moreover, trigeminal neurons innervating intracranial arteries in rats have been shown to be more frequently CGRP positive than neurons innervating the forehead or mandible [52]. CGRP positive fibers are localized to the tunica adventitia in cerebral arteries, and to the border of the tunica media [7]. CGRP positive nerve fibers also innervate the dura mater, where the receptor is specifically found in nerve bundles, in the smooth muscle layer of arterial vessels and in mononuclear cells (probably mast cells) localized in close proximity to the arteries [53].

## 3. CGRP Receptors

**Figure 2:**
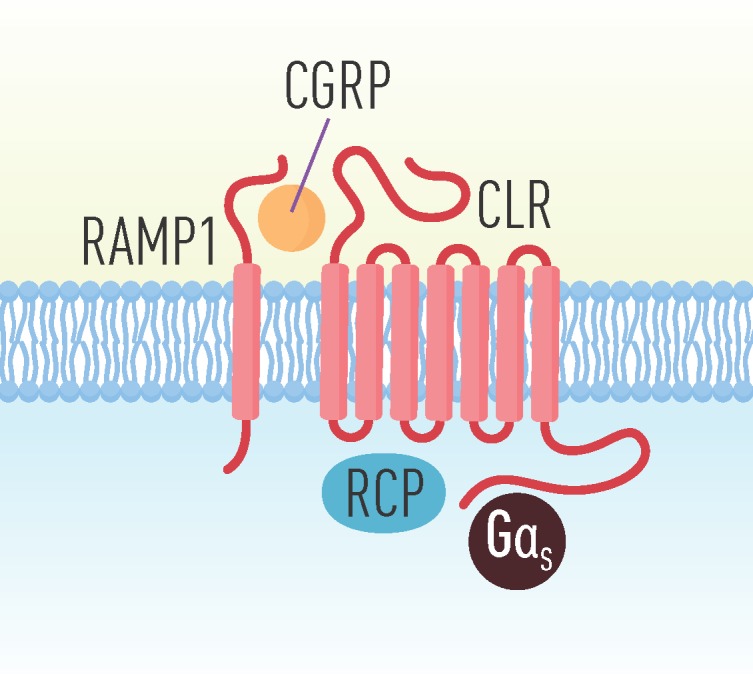
The CGRP receptor complex which consists of a G protein-coupled receptor called calcitonin receptor-like receptor (CALCRL), and a single transmembrane domain protein receptor, receptor activity-modifying protein (RAMP1) and two cytoplasmic proteins, receptor coupling protein (RCP) and the α-subunit of the GS protein (Gα_S_).

CGRP mediates its effects through the CGRP receptor, a heteromeric receptor complex illustrated in Figure 2. The CGRP receptor is composed of two transmembrane proteins; a G protein-coupled receptor called CALCRL, and a single transmembrane domain protein receptor, RAMP1. This heterodimer is coupled to two cytoplasmic proteins; receptor coupling protein (RCP) and the α-subunit of the GS protein (Gα_S_) [54,55]. Both the α- and β-CGRP isoforms are complete agonists of the receptor [56].

The main functional unit of the receptor, CALCRL (also known as CLR or CRLR), can be linked to one of three RAMPs which, respectively, produces different receptors [57]. RAMPs are single-transmembrane proteins pivotal for receptor function, as they are involved in the translocation of the receptor complex to the plasma membrane, and further provides the specificity for ligand binding [57,58]. With RAMP1, CALCRL forms the CGRP receptor, with RAMP2 an AM receptor, designated AM1 and with RAMP3, a dual CGRP/AM receptor designated AM2 [59]. The RAMP1 subunit is responsible for specific binding of CGRP to the CGRP receptor [60,61]. However, CGRP is proposed to bind to all of these receptor complexes with different affinity [62]. In addition, RAMP1-3 forms three receptors for amylin (AMY1-3) by the heterodimeric association to the calcitonin receptor (CTR), whereof AMY1 has an equal affinity with amylin and CGRP [56,63]. The vasodilation of cranial arteries and the associated pain that typically occurs during a headache attack is hypothesized to be the result of CGRP release [59]. The transition from a physiological event to a pathological one could potentially be the result of a malfunctioning receptor, altered CGRP binding or signaling at the receptor level, making RAMP1 an interesting pharmacological target. To support this hypothesis, mice overexpressing RAMP1 in the nervous system display increased aversion to light and are furthermore hypersensitive to mechanical allodynia [64,65].

The last component needed for a functional CGRP receptor is the receptor component protein (RCP). Lack of RCP has been shown to affect activation of the CGRP receptor, measured as an increase in cyclic adenosine monophosphate (cAMP), while not affecting ligand binding, leading to the assumption that RCP association with the CGRP receptor is required for signal transduction through the Gα_S_ [66]. Gα_S_ stimulates the cAMP-dependent pathway by activating adenylyl cyclase.

CGRP signaling includes the regulation and desensitization of its receptor following the activation by an agonist [67]. When CGRP has bound to its receptor, CALCRL is quickly phosphorylated, which leads to internalization of the receptor via recruitment of β-arrestins [68]. Depending on the temporal characteristics of the activation, they are either recycled back to the cell membrane or degraded [69]. Transitory receptor activation leads to internalization, which is followed by receptor recycling back to the surface of the cell and resensitization, while continuous activation leads to internalization and receptor degradation. In a study by Manoukian et al. tool antibodies against CGRP and its receptor were used to estimate CGRP receptor internalization and cAMP production using fluorogen-activated protein technology in a novel cellular model [70]. This model showed that the antagonist antibodies blocked the CGRP-induced cAMP signaling, as well as the CGRP receptor internalization. In addition, it was shown that at least 10-fold higher concentrations of either antibody is necessary to block CGRP receptor internalization compared with cAMP accumulation.

CGRP signaling is known to be affected in vascular, inflammatory, as well as neurological diseases [20,71,72,73]. There is an unusually high level of complexity to these signaling pathways. In view of the high similarity of the calcitonin family of peptides and the possible cross-reactivity with associated receptors, this warrants a more thorough investigation of the receptor proteins to increase the understanding of CGRP signaling and its implication in disease.

## 4. CGRP Function

CGRP was initially described as a vasodilator, and the dilatation was associated with an increase in cAMP and independent of endothelial function [9]. This view has developed, and CGRP is now considered as a multifunctional regulatory agent. Nevertheless, CGRP is reported to be one of the most efficient microvascular vasodilator substances, with a potency 10–100 times greater than other known vasodilators (e.g., acetylcholine and substance P) [74].

Previous studies report that serum histamine levels in whole blood during a CH attack is increased which indicates activation of mast cells [75]. This is further supported by reports on the peripheral release of CGRP, which trigger mast cell degranulation and contributes to neurogenic inflammation together with substance P [76,77]. In addition, CGRP receptors have been identified in dural mast cells [53].

Presynaptic terminals in nerves that produce CGRP, take the form of focal swellings, occurring at regular intervals along the axon [23]. This mechanism may occur at the molecular level via axon-axon modulation of the two types of sensory fibers [78]. The C-fibers may modulate adjacent Aδ-fibers through axon-axon CGRP signaling at nodes of Ranvier in the trigeminal system. When the nerve is stimulated, CGRP is released from its storage via calcium (Ca^2+^)—dependent exocytosis. Moreover, CGRP can be released via a Ca^2+^ independent mechanism mediated by activation of acid-sensitive ion channels, or by secretion directly from the cell body with the aim of auto-amplifying the CGRP signaling, as well as activation of surrounding neurons and glia [79,80]. The synaptic release of CGRP is regulated by neuronal presynaptic receptors. These presynaptic receptors are serotonin (5-hydroxytryptamine) receptor 5-HT1B and 5-HT1D, which inhibit CGRP release [81]. Triptans effectively alleviate migraines in many patients by acting on these two serotonin receptors [23].

The vasodilation caused by CGRP release is the result of signal transduction via the protein kinase A (PKA) pathway. PKA is activated by an increase of cAMP and can further lead to a multitude of physiological responses, such as pain perception, production of nitric oxide (NO), activation of Ca^2+^ channels, etc. via several downstream effector proteins [59]. There is some evidence that CGRP receptors can signal via Gα-coupled proteins other than Gα_S_; these pathways are distinguished from PKA mediated responses by the lack of increase in cAMP [82]. For example, the activation of the c-Jun N-terminal kinase (JNK) pathway via PKC activation or Gα_i/o_ receptor coupling has been suggested [83,84]. Phospholipase C β1 could potentially act as a CGRP effector protein following receptor coupling with Gα_q/11_. This pathway results in an increase in Inositol trisphosphate (IP3) and activation of Ca^2+^ channels in the endoplasmic reticulum (ER) with subsequent Ca^2+^ release [85]. It should be noted that the observed effect could equally be the result of calcitonin signaling via the AMY1 receptor [86]. Since many studies have been performed in cell lines or transfected cells, results should be interpreted with care. In vivo, the activation of different effector proteins might vary with cell type, and could potentially also occur simultaneously.

## 5. CGRP and Primary Headache

The dilation of cranial arteries arising during headache attacks is a suggested consequence of CGRP release [17]. Today CGRP has been demonstrated to be the major neuronal messenger associated with headache in migraine and CH [20,71]. Numerous studies have shown a clear link between differences in CGRP levels and headache bouts, including CH [20,22,87,88,89,90].

### 5.1. Migraine Pathophysiology and CGRP

Intravenous infusion of CGRP has previously shown to induce a delayed, migraine-like headache in 57–75% of migraine patients with and without aura [91,92,93]. Because CGRP did not have this effect on healthy controls, it was suggested that migraine patients had increased sensitivity to CGRP [93]. However, this is not a general phenomenon. Local peripheral administration of CGRP in migraine patients and matched controls did not show a difference in microcirculatory dilatation [94]. In human subjects, local forehead administration of substance P elicited pain, while CGRP did not [95,96].

CGRP is reported to be elevated in external jugular venous blood during a migraine attack, but not in the peripheral cubital fossa blood [20,71,97,98]. Other studies have suggested that CGRP might be elevated in peripheral blood of migraine patients when they are not experiencing a migraine attack [99]. Much of this discussion is related to the methodology of performing the experiments; some problems and pitfalls have been discussed [97]. CGRP levels have also been reported to be elevated in tears fluid and saliva in migraine patients compared to healthy controls [100,101].

Further evidence for CGRP having a central role in headache has been demonstrated by studies showing that successful treatment of migraine pain with the serotonin 5-HT1B/1D receptor agonist sumatriptan, and other ‘triptan’ drugs, resulted in the normalization of CGRP levels [98]. Sumatriptan causes increased intracellular Ca^2+^, which, in turn, repress CGRP promoter activity [102].

### 5.2. Cluster Headache Pathophysiology and CGRP

**Figure 3:**
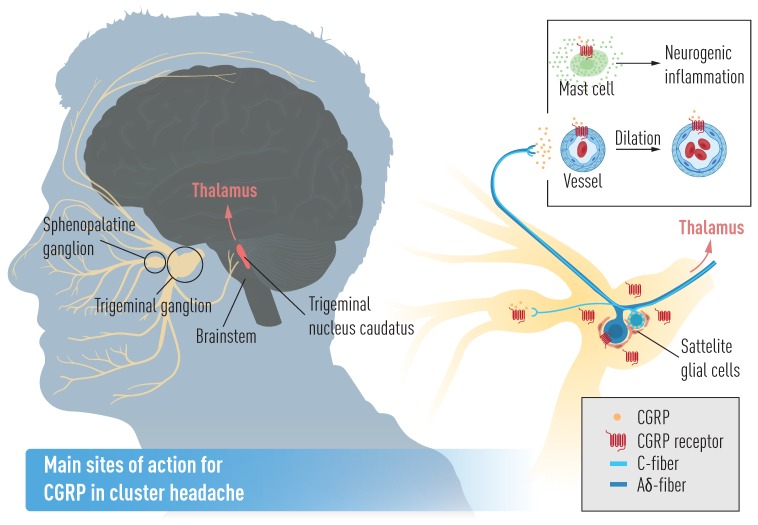
Main sites of action for CGRP in cluster headache. CGRP originates in the trigeminal ganglion with sensory C- and Aδ-fibers that project to various parts of the face and head, as well as to intracranial structures.

CGRP and all the CGRP receptor components are prominent in many parts of the trigeminovascular system (Figure 3) [17], which has been shown to be activated during CH attacks [20,103]. During CH attacks the trigeminal-autonomic reflex (an association between the trigeminal sensory system and the parasympathetic system—sphenopalatine and otic ganglia) is activated provoking vasodilation of cranial arteries by the release of vasodilatory molecules, including CGRP, vasoactive intestinal peptide (VIP) and pituitary adenylate cyclase-activating polypeptide (PACAP) [20,104]. In addition, a recent study shows that CGRP infusion in CH patients in active period triggers attacks, but not in remission [21]. Interestingly, chronic CH patients were discovered to have lower plasma levels of CGRP than episodic CH patients, although very little is known on how the pathophysiology differs between these two conditions [90].

## 6. CGRP and Genetics

So far, there has been very little focus on the genetic aspects of CGRP and its receptors in CH. A small GWAS analysis of 99 CH patients did not reveal any associations with CGRP-related genes, but suggested implication of the PACAP receptor 1 (PAC1 receptor) gene (ADCYAP1R1) [105]. PACAP, present in a subpopulation of neurons in the trigeminovascular system, is presumed to be involved in headache pathophysiology and resembles CGRP to some degree [15,106]. However, PACAP, as well as VIP, are both expressed in most of the parasympathetic ganglia neurons, while CGRP is localized to fibers [107,108]. The association between CH and ADCYAP1R1 could not be confirmed in an independent larger study [109]. One genetic variant in RAMP1 has been successfully linked to CH through a candidate gene study in a Swedish cohort [110]. A significant difference in the RAMP1 single nucleotide polymorphism rs3754701 frequency was identified between Swedish CH patients and controls. Moreover, RAMP1 polymorphisms have previously been suggested to be implicated in both migraine and medication overuse headache [60,61], and the associated SNPs, rs3754701 and rs7590387, are part of a risk haplotype for cerebral infarction reported in a Japanese case-control study [111]. In addition, RAMP1 mRNA expression was shown to be enhanced in primary fibroblasts from CH patients compared to controls [110]. These findings suggest the involvement of the RAMP1 gene in the CH pathophysiology and further studies are wanted in order to confirm this hypothesis. There are so far only three published studies on broad screenings for genes differentially transcribed between individuals with CH and controls [112,113,114]. Neither of these three studies reports differences in gene expression of CGRP or any of its receptor components. However all three studies have relatively low power to detect small differences in gene expression after correction for multiple testing, due to low sample size in relation to high number of tests (Costa et al. [112], *n* = 8 (CH) and *n* = 10 (controls) vs. 36,079 transcripts, Sjöstrand et al. [113] *n* = 3 (CH) and *n* = 3 (controls) vs. 54,000 gene transcripts and Eising et al. [114] *n*  =  19 (episodic CH) and *n*  =  20 (chronic CH) patients and *n*  =  20 (controls) vs. 13,416 genes).

## 7. CGRP and Treatment

CGRP and the CGRP receptor have recently taken center stage as therapeutic targets for primary headaches [115]. Multiple CGRP and CGRP receptor antibodies, as well as antagonists, constitute a new frontier of migraine and CH medication [116,117,118,119].

### 7.1. Migraine Treatment

On 17 May 2018, the U.S. Food and Drug Administration approved the first CGRP receptor antibody, Aimovig^®^ (erenumab-aooe) (Amgen, Thousand Oaks, CA, USA), for the preventive treatment of migraine in adults [14]. CGRP preventive treatment is now being revolutionized after the licensing of in total three monoclonal antibodies (MABs) directed towards the CGRP ligand or its receptor, erenumab, fremanezumab, and galcanezumab, which are effective and well-tolerated. The monoclonal antibodies against CGRP (eptinezumab, fremanezumab and galcanezumab) and the CGRP receptor (erenumab) effectively prevent migraine attacks [23]. Further characterization of erenumab has demonstrated that it specifically inhibits CGRP-induced relaxation of cranial arteries without impacting vasodilatory responses or contractile responses of endogenous (e.g., substance P) or pharmacological (e.g., sumatriptan) vasoactive compounds [120]. New acute therapies for migraine include small molecule CGRP receptor antagonists, gepants [121]. These antagonists lack active vasoconstrictor effect [49,122,123,124]. To this date, six gepants have been tested, and each was reported effective in the acute treatment of migraine [121]. Two were, however, terminated during development, due to suspected hepatic damage, but this has been reported to be based on metabolites not on the CGRP mechanism and two gepants, rimegepant and ubrogepant, have completed Phase III clinical trials.

### 7.2. Future CH Treatment

The CGRP monoclonal antibody galcanezumab (Emgality^®^, Eli Lilly, Indianapolis, IN, USA), has been reported effective and well-tolerated in a placebo-controlled trial for preventive treatment of episodic CH in Phase III [125]. One hundred and six patients were enrolled in the study, where 49 (age 47 ± 11 years) were randomly assigned to receive galcanezumab and 57 (age 45 ± 11 years) to receive a placebo. Safety evaluation was performed, including the assessment of spontaneously reported adverse events, electrocardiograms, vital signs, and laboratory measures. Galcanezumab was administered subcutaneously to patients with episodic CH, which reduced the weekly frequency of attacks across weeks 1 through 3 after the initial injection, compared to the placebo group. The galcanezumab dose was 300 mg dose once a month which can be compared with 240 mg/month followed by 120 mg/month for migraine [126]. The mean reduction in the frequency of attacks across weeks 1 through 3 was 8.7 attacks in the galcanezumab group, compared with 5.2 in the placebo group (95% CI, 0.2 to 6.7; *p* = 0.04). The proportion of patients with a reduction of at least 50% in headache frequency at week 3 was 71% in the galcanezumab group and 53% in the placebo group. During the double-blind phase of the trial, no deaths or serious adverse events occurred. A higher frequency of adverse events was observed in the galcanezumab group than in the placebo group (43% vs. 33%), a majority of these events were rated mild to moderate in severity. In June 2019, galcanezumab got FDA Go-Ahead for episodic CH.

The site of action of galcanezumab is currently unknown, and it is known that IgG antibodies enter the cerebrospinal fluid at only 0.1% of the plasma concentration [127]. This may suggest a peripheral site of action, such as the trigeminal ganglion. The trigeminal ganglion is not protected by the blood-brain barrier (BBB) as demonstrated by dye uptake in the trigeminal ganglion after intravenous injection with Evans blue [44], as well as using a quantitative method for calculation of the “permeability-surface index” of the BBB [128].

Aimovig has not been FDA-approved to prevent CH, but it may be tested off-label for this purpose [129]. However, it is not currently known if Aimovig is effective at preventing CH. A clinical trial of fremanezumab (Ajovy^®^, Teva, Petah Tikva, Israel) in chronic CH, was stopped at an early stage by the drug manufacturer because Ajovy did not reduce the number of headaches for individuals included in the study during a specific time frame [130,131]. Teva has since then stopped all clinical trials on Ajovy as a treatment for CH, after failing to meet the primary endpoint also in patients with episodic CH [130,132]. The open-label extension studies of galcanezumab (NCT02797951) are ongoing and are expected to end in 2020 [133]. There is so far no study reported on eptinezumab for CH prevention, nor on the usage of gepants for acute treatment of CH.

To summarize, the first clinical trials on CGRP antibodies as a treatment for CH has so far shown a moderate effect on episodic CH, but failed to relieve symptoms in chronic CH patients. Considering the efficiency of anti-CGRP treatment in migraine, and the clear involvement of CGRP in CH pathophysiology, these results were somewhat unexpected.

It is possible that the treatment protocol has to be adjusted in chronic CH patients, and that an effect would be visible using, e.g., other treatment intervals, doses or lengths. Moreover, since the manifestation of symptoms is periodic in CH and CGRP sensitivity is normal in CH patients outside of a bout, CGRP monoclonal antibodies might only be effective in certain windows of the disease [21]. Perhaps anti-CGRP treatment should be considered a CH modifying treatment, used for example to interrupt the development of a new active phase, or to prevent chronification of symptoms. It is known that CH patients can switch phenotype from episodic to chronic and vice versa. If there is indeed a permissive window where the patient is susceptible to anti-CGRP, this window can also be the key to the lack of response in chronic CH patients, as they might have reached another “non-responsive” physiological state. For example, chronic CH patients were found to have lower plasma CGRP levels than episodic CH patients in remission in a recent Danish study [90]. Current anti-CGRP treatments are presumed to act on peripheral targets as monoclonal antibodies do not cross the blood-brain barrier. There is the possibility of a direct central mechanism of CGRP signaling in CH, involving central mechanisms of pain sensitization. Whether or not CGRP is acting on a central level in headache pathophysiology will become clearer with the development of gepants as they can circulate more freely across the blood-brain barrier, and therefore, reach new drugable targets.

## 8. Conclusions

CGRP is involved in CH pathophysiology, and there is, in addition, a genetic link to the CGRP receptor complex. CGRP and the CGRP receptor have taken center stage as therapeutic targets for primary headaches, and multiple CGRP and CGRP receptor antibodies constitute a new frontier of headache medication. One of the CGRP monoclonal antibody galcanezumab, has been reported effective and well-tolerated in a placebo-controlled phase III trial for preventive treatment of episodic CH and got an FDA approval in June this year. The missing effect of galcanezumab in chronic CH might be due to a need to adjust treatment protocol using, e.g., other treatment intervals, doses or lengths to have a visible effect in chronic CH patients. Reports on CGRP in relation to CH suggest involvement in CH pathophysiology and further studies are wanted in order to get a deeper knowledge in the field.
